# Efficient Visual Search from Synchronized Auditory Signals Requires Transient Audiovisual Events

**DOI:** 10.1371/journal.pone.0010664

**Published:** 2010-05-14

**Authors:** Erik Van der Burg, John Cass, Christian N. L. Olivers, Jan Theeuwes, David Alais

**Affiliations:** 1 Department of Cognitive Psychology, Vrije Universiteit Amsterdam, Amsterdam, The Netherlands; 2 School of Psychology, University of Western Sydney, Sydney, Australia; 3 MARCS Auditory Research Laboratories, University of Western Sydney, Sydney, Australia; 4 School of Psychology, University of Sydney, Sydney, Australia; Ludwig Maximilians University Munich, Germany

## Abstract

**Background:**

A prevailing view is that audiovisual integration requires temporally coincident signals. However, a recent study failed to find any evidence for audiovisual integration in visual search even when using synchronized audiovisual events. An important question is what information is critical to observe audiovisual integration.

**Methodology/Principal Findings:**

Here we demonstrate that temporal coincidence (i.e., synchrony) of auditory and visual components can trigger audiovisual interaction in cluttered displays and consequently produce very fast and efficient target identification. In visual search experiments, subjects found a modulating visual target vastly more efficiently when it was paired with a synchronous auditory signal. By manipulating the kind of temporal modulation (sine wave vs. square wave vs. difference wave; harmonic sine-wave synthesis; gradient of onset/offset ramps) we show that abrupt visual events are required for this search efficiency to occur, and that sinusoidal audiovisual modulations do not support efficient search.

**Conclusions/Significance:**

Thus, audiovisual temporal alignment will only lead to benefits in visual search if the changes in the component signals are both synchronized and transient. We propose that transient signals are necessary in synchrony-driven binding to avoid spurious interactions with unrelated signals when these occur close together in time.

## Introduction

Many objects and events in our natural environment produce complementary light and sound energy that can be detected by specialized visual and auditory sensory systems. This information may be integrated across sensory modalities to take advantage of informational redundancy, enhancing the representation of the external world and making it more robust [1], [2]. A picture of the neural mechanisms underlying the processes of multisensory integration is emerging [3], [4] to complement the many examples of integrated audiovisual information exerting a powerful influence over various aspects of perception [5], [6], [7], [8], [9], [10]. Most studies found evidence that vision tends to dominate audiovisual perception for spatial tasks [11], [12], and audition tends to dominate for temporal tasks [9], [13], [14]. However, this tendency is flexible and can be reversed when information in a dominant sense is degraded [6].

Many of the studies examining audiovisual perception have used sparse displays, often involving a single stimulus. While this approach has been sufficient to demonstrate that audiovisual interactions do occur and can influence spatial and temporal aspects of perception, it is more relevant to real world contexts to examine how audiovisual interactions affect our ability to locate and identify objects in cluttered environments. In such cases, there are typically many objects distributed across space that compete for and distract our attention. Two studies, both involving speeded visual search, investigated this question. Fujisaki, Koene, Arnold, Johnston & Nishida [15] found that the time required for observers to identify whether a temporally modulating visual singleton was synchronised with an amplitude or frequency modulating auditory tone increased precipitously with the number of distractors present. Such “serial search” performance suggests that the analysis of audiovisual temporal events is not automatic and pre-attentive but rather requires effortful, cognitive analysis. In stark contrast to this null finding, Van der Burg, Olivers, Bronkhorst & Theeuwes [16] found that an abrupt (and non-spatial) auditory pulse synchronised with an abrupt color change of a visual singleton strongly facilitated the speed of target identification, and did so independently of the number of distractor elements.

Why do Van der Burg and colleagues find evidence for such audiovisual interaction whereas Fujisaki, et al. do not? The purpose of this paper is to identify what information is critical for a non-spatial acoustic signal to drive efficient visual search. To preview our results, we find that efficient search is contingent upon visual luminance changes being abrupt and uniquely synchronised with the auditory event. Our results demonstrate that audiovisually synchronised sine-wave modulations do not facilitate efficient visual search, while synchronised square-wave modulations do (Experiment 1). Experiment 2 shows that this acoustically driven visual search efficiency requires abrupt (square-wave) luminance modulations, although auditory events can be sinusoidal. We also show that visual search efficiency improves monotonically with the number of odd harmonics added to the fundamental sine-wave (progressively approximating a square-wave), although the harmonics presented alone afford no search benefit (Experiment 3). Furthermore, we show that these search benefits are not due to the dynamic properties of the surrounding distractors, but due to an increase in audiovisual onset and/or offset slope (Experiment 4). In sum, the benefit of square-wave compared with sine-wave modulation is not due to continuous vs. discrete variation or to amplitude differences but to the presence of a transient luminance event synchronised with an auditory stimulus.

## Results

**Figure 1:**
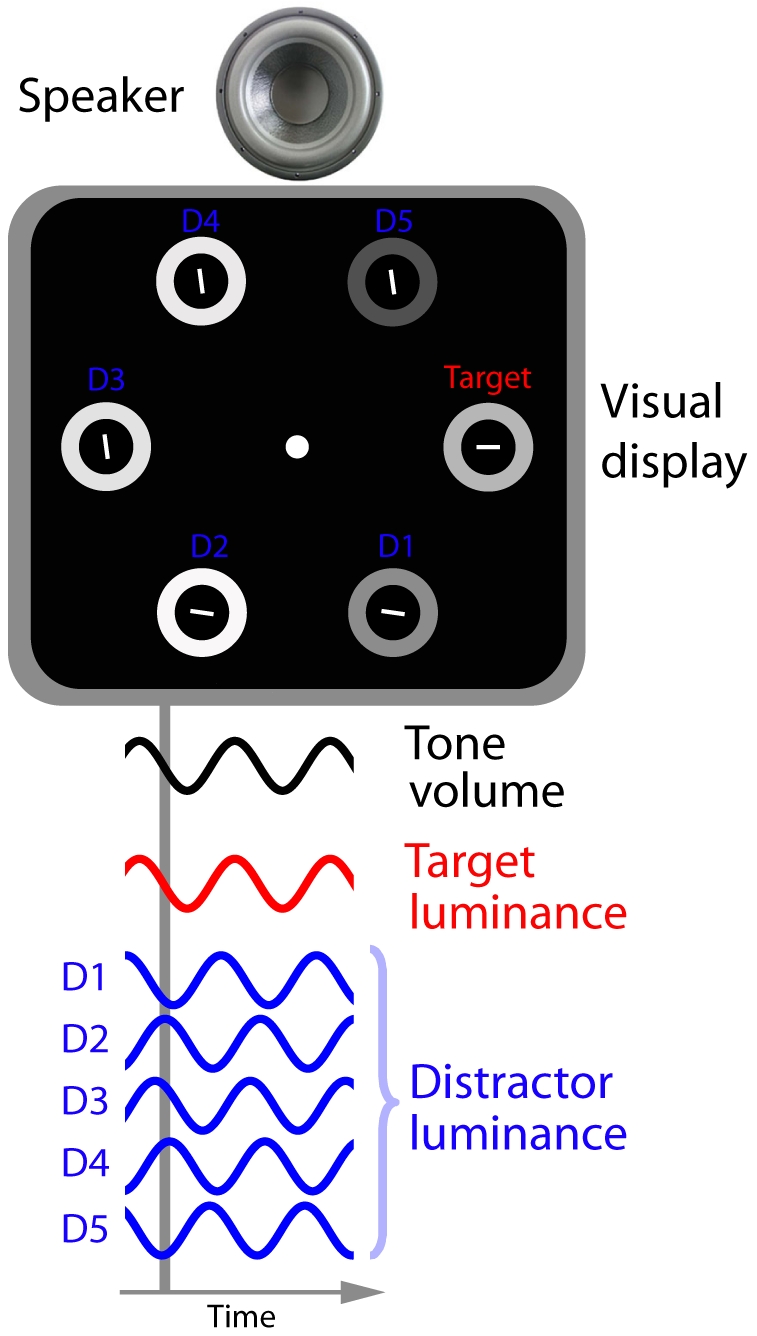
Illustration of an example search display. Participants were asked to make speeded responses toward the orientation of a visual target (a horizontal or vertical line segment). All line segments were surrounded by annuli, whose luminance changed continuously during a trial. On half of the trials the luminance of all annuli followed a sine-wave pattern (as shown in the figure), and on the remaining trials the annuli followed a square-wave pattern. If present, a spatially uninformative tone was synchronized with the target annulus (i.e. the one surrounding the target element). See Movie S1 and Movie S2 for examples of sine-wave and square-wave trials, respectively.

In the present study, participants were asked to make a speeded response regarding the orientation of a visual target (a horizontal or vertical line segment) among 5 or 10 distractor lines of various orientations, by pressing the z- or m-key when they detected a horizontal or vertical line segment, respectively. Figure 1 illustrates an example search display used in the present study.

### Experiment 1: Sine-wave vs. square-wave audiovisual events

All line segments were surrounded by annuli whose luminance modulated continuously during each trial. On half of the trials, the luminance of all annuli modulated with a sine-wave temporal profile (Movie S1; fundamental frequency  = 0.72 Hz), and on the remaining trials the annuli modulated with a square-wave profile (Movie S2; again 0.72 Hz). On half of the trials, the target annulus (i.e. the one surrounding the horizontal or vertical line segment) was either synchronized with an auditory signal whose intensity had an identical temporal modulation profile to the target annuli (i.e. both were perfectly aligned in phase), or was desynchronized by shifting the auditory signal 180° out of phase with the target annuli modulation. In the remaining half of trials the search display was presented in silence. Note that the distractor annuli were never in phase with the tone.

**Figure 2:**
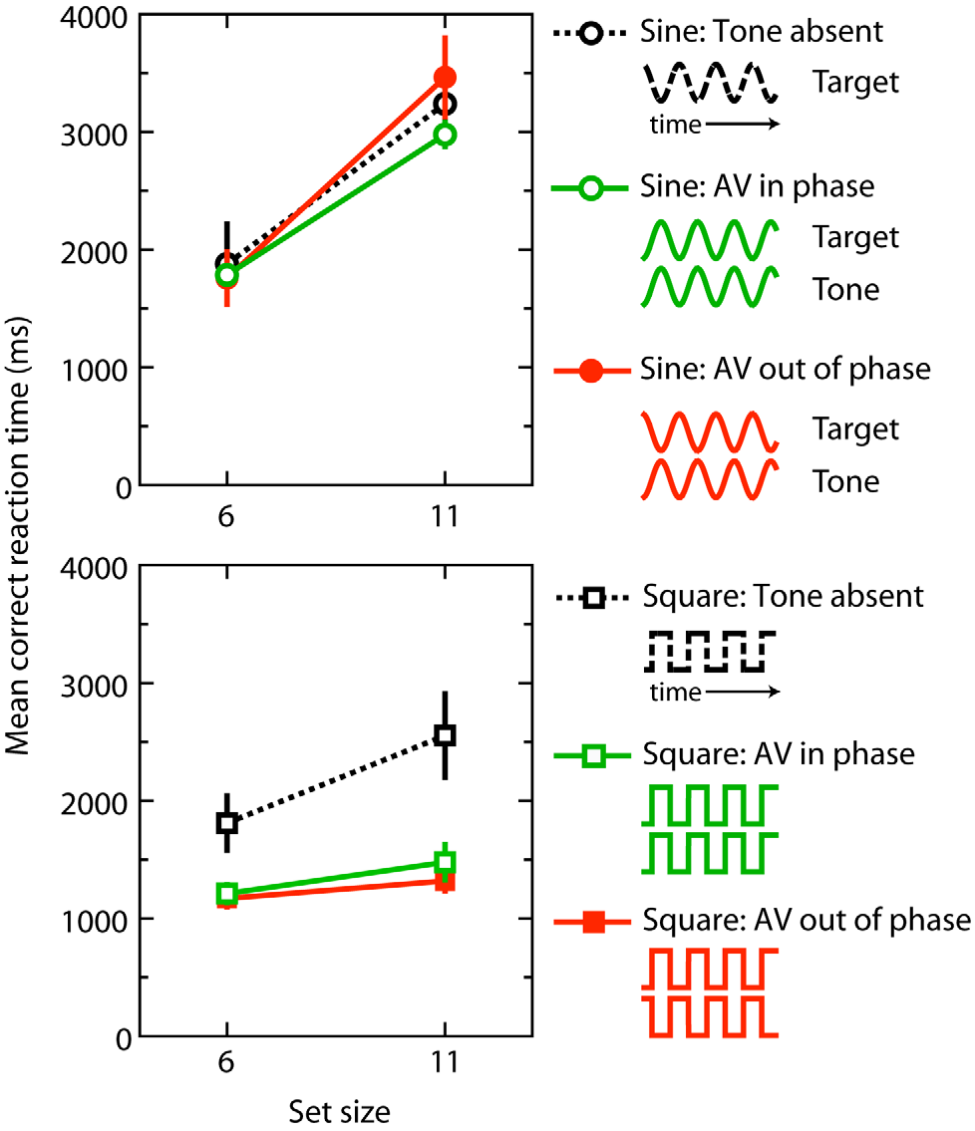
Results of Experiment 1. Mean correct reaction time as a function of set size, stimulus type and tone presence. The mean correct RT reflects the time between the search display onset and the time to respond (correctly) to the target orientation. The error bars reflect the overall standard errors of individuals' mean RTs.

The results of Experiment 1 are presented in Figure 2. Reaction time (RT) data from practice blocks and erroneous trials were excluded. RTs were subjected to a repeated-measures Univariate Analysis of Variance (ANOVA) with set size (6 vs. 11), tone presence [present vs. present (180° out of phase) vs. absent] and stimulus type (sine- vs. square-wave) as within-subject variables.

Overall mean error rate was low (1.6%) and was therefore not subjected to further analysis. The ANOVA on RTs revealed a significant three-way interaction among set size, tone presence and stimulus type, *F*(2, 8) = 5.3, *p*<.05. This interaction was further examined by separate ANOVAs for each stimulus type. When the audiovisual events were square-waves, the ANOVA yielded a reliable tone presence x set size interaction, *F*(2, 6) = 9.5, *p* = .01, indicating that search improved when a tone was synchronized with the target annulus (search slope was 38 ms item^−1^ when the tone was in phase; search slope was 42 ms item^−1^ when the tone was 180° out of phase) compared to the condition that no such tone was present (162 ms item^−1^). In contrast, when the audiovisual events were sine-wave modulated, no such audiovisual interaction was observed, *F*(2, 6) = 1.7, *p* = .257. Neither was the main effect of tone presence reliable, *F*<1.

The findings of this experiment are clear. Synchronized sinusoidal audiovisual events did not lead to any search benefits whatsoever [15]. In contrast, synchronized square-wave audiovisual modulations (with the same fundamental temporal frequency as the sine-waves) produced a potent facilitation in visual search performance [16].

The substantial search slopes in the present experiment might suggest that the visual event did not capture attention in an automatic fashion. Even though this is correct, we would like to note that, in contrast with classic visual search studies (in which the salient singleton is always visible until the response is made [17]), the salient event in the present case (i.e. the synchronized audiovisual event) is only temporarily present. As a result, if participants miss this salient event, then they have to wait for the next opportunity (i.e. the subsequent synchronized audiovisual event). This is consistent with our previous studies [16], [18], in which we have found evidence that synchronized audiovisual events capture attention on most, but not on all trials. A feasible explanation for these misses might be attributed to eye-movements, and blinks [16].

Even though we observed substantial search benefits in the square-wave condition, the audiovisual events were always simultaneously presented (i.e. the audiovisual events were fully synchronized without a delay), which might not have been optimal for the sinusoidal condition. For instance, several studies report that to perceive audiovisual simultaneity, the auditory event needs to be presented after the visual event by about 30–40 ms [19], [20], [21], [22]. One potential reason for our effect of stimulus type might be that the sine-wave condition is more sensitive to temporal synchrony than the square-wave condition. Therefore, in a control experiment (Experiment S1), we manipulated the synchronization of the audiovisual events, by varying the time between the tone and the target (tone-target interval; TTI). We observed optimal search performance in the square-wave conditions when the auditory signal was presented 40 ms after the visual event [19], [20], [21], [22]. In contrast, no effect of TTI was observed when audiovisual events were sinusoidally modulated (see Figure S1).

An interesting feature of these results is that the synchronised sinusoidal modulating AV stimulus generates a greater proportion of unique correlations over time between luminance and intensity changes than do synchronised square-wave modulation. Therefore, one might argue that the sinusoidal auditory event contains more temporal information about the luminance of the synchronized annulus than the square-wave modulating AV stimulus. Despite this, sinusoidal modulating audiovisual search was far less efficient than square-wave modulation. Moreover, search performance in the in-phase square-wave condition was equal to the search performance in the out of phase square-wave condition, also suggesting that participants did not adopt a strategy to search for a specific luminance change. The data suggest that auditory-driven visual search may require an abrupt temporal event, which was present when the audiovisual events were square-wave modulated (regardless of whether the auditory and visual modulations were in-phase or in counter-phase), but not when the audiovisual events were sinusoidally modulated. In the next experiment we investigate whether auditory driven improvement in visual search efficiency is contingent upon an abrupt and unique change in the auditory modality, the visual modality, or perhaps both modalities.

### Experiment 2: A special role for visual transient signals

In Experiment 2, we independently manipulated the auditory stimulus type (sine- vs. square-wave), and the visual stimulus type (sine- vs. square-wave). In contrast with the previous experiment, the tone was always present, and always synchronized with the target annulus (i.e. in phase). Auditory stimulus type (sine- vs. square-wave) and visual stimulus type (sine- vs. square-wave) were randomly mixed within blocks. Participants received ten experimental blocks of 32 trials, preceded by a single practice block of 32 trials. If audiovisual synchrony in cluttered displays requires temporally abrupt signals in both modalities, then we expect to observe search benefits when both are square-wave modulated, but not when either one is sinusoidaly modulated.

**Figure 3:**
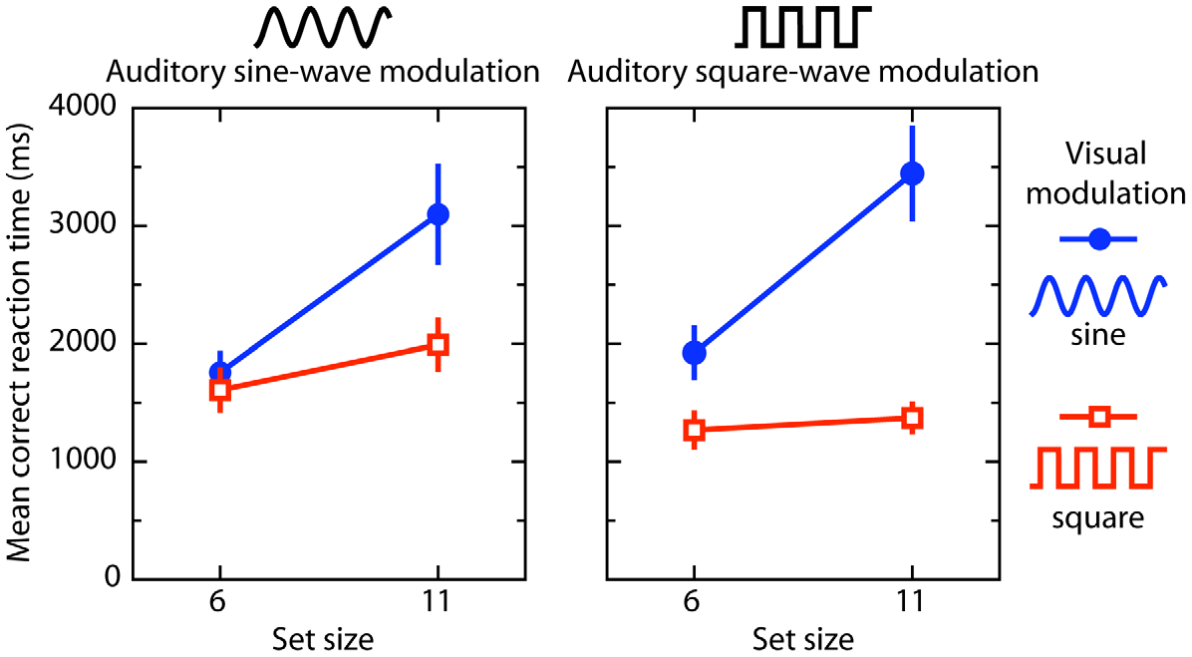
Results of Experiment 2. Mean correct reaction time as a function of set size, auditory stimulus type and visual stimulus type. The data show that auditory driven visual search efficiency requires square-wave visual onsets.

The results of Experiment 2 are presented in Figure 3. RTs were subjected to an ANOVA with set size (6 vs. 11), auditory stimulus type (sine- vs. square-wave), and visual stimulus type (sine- vs. square-wave) as within-subject variables.

Overall mean error rate was 2.0%. The ANOVA on RTs revealed a reliable three-way interaction among auditory stimulus type, visual stimulus type and set size, *F*(1, 3) = 18.3, *p*<.05. This interaction was further examined by separate ANOVAs for each auditory stimulus type. When the auditory signals were sine-waves, the ANOVA yielded a reliable visual stimulus type x set size interaction, *F*(1, 3) = 13.6, *p*<.05, as the search slopes were shallower when the visual annuli were square-waves (113 ms item^−1^) than when the visual annuli were sine-waves (276 ms item^−1^). When the auditory signals were square-waves, the ANOVA revealed a similar visual stimulus type x set size interaction, *F*(1, 3) = 121.9, *p*<.005, as the search slopes were shallower when the visual annuli were square-waves (23 ms item^−1^) than when the visual annuli were sine-waves (326 ms item^−1^). The interaction was also further examined by separate ANOVAs for each visual stimulus type. When the visual annuli were square-waves, search slopes were shallower when the auditory signals were square-waves (23 ms item^−1^) than when the auditory signals were sine-waves (113 ms item^−1^), *F*(1, 3) = 40.5, *p*<.01. In contrast, no such interaction was observed when the visual annuli were sine-waves, *F*(1, 3) = 1.8, *p* = .270.

Together, these data provide compelling evidence that acoustically driven visual search efficiency requires abrupt (square-wave) visual onsets, although auditory events can be sinusoidal. However, search performance was optimal when both the synchronized audiovisual events were square-wave modulated.

### Experiment 3: Harmonic sine-wave synthesis

**Figure 4:**
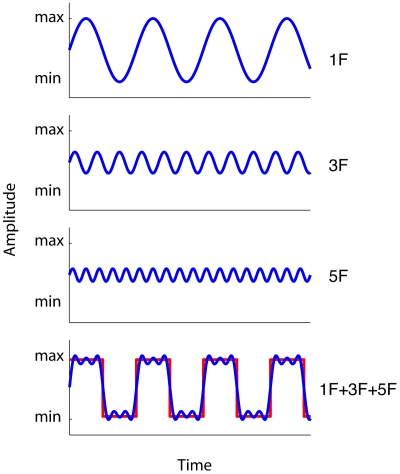
Illustration of the first three odd harmonics of the square-wave (with F the fundamental frequency). The blue line (lower panel) indicates a square-wave that results from summating the first three odd higher harmonics. The red line indicates a square-wave that would result from summating an infinite number of odd harmonics.

The previous experiments showed that audiovisually synchronised sine-wave modulations do not facilitate efficient visual search, while synchronised square-wave modulations do. The difference between a sine- and square-wave can be mathematically derived by harmonic synthesis. This refers to the fact that waveforms can be constructed by adding together sine-waves of the appropriate amplitudes, frequencies and phases. For instance, a square-wave with a frequency of 0.72 Hz, can be easily created by adding higher odd (phase aligned) harmonics to the fundamental sine-wave (F) with a frequency of 0.72 Hz. Figure 4a shows the first three odd harmonics used to synthesize a square-wave (the blue line results from the sum of the three harmonics). The red line indicates a square-wave that results from summating the infinite number of odd harmonics.

In the present experiment, we tested whether adding higher odd harmonics (3F, 5F and 7F) to the audiovisual fundamental frequency (F) used in Experiments 1 and 2 (0.72 Hz), progressively approaching a square-wave, would affect visual search. We also conducted a control experiment, in which we investigated whether individual higher odd harmonic components affect visual search when presented in isolation (i.e. unsynthesized) compared to the sum of the higher odd harmonics. The experiment was identical to Experiment 2, except that the waveforms defining auditory and visual modulation were always identical. Furthermore, we systematically manipulated the number of higher odd harmonics (3F, 5F, 7F) added to the fundamental frequency (F). See Movie S3 for an example trial in which the fundamental frequency (F) was accompanied by the third and fifth harmonic (F+3F+5F). We also included a condition in which the number of odd harmonics was infinite (square-wave condition, within Nyquist limits). Stimulus type [fundamental (F), F+3F, F+3F+5F, F+3F+5F+7F, or square-wave] was randomly mixed within blocks. Participants received ten experimental blocks of 40 trials, preceded by a single practice block of 40 trials.

The control experiment was identical, except that stimulus type contained the individual odd harmonics (F, 3F, 5F, or 7F), or the sum of all harmonics (F+3F+5F+7F). Stimulus type was randomly mixed within blocks. Participants participated in two sessions. One session contained one practice block and ten experimental blocks of 40 trials each. If auditory-driven visual search follows harmonic principles, then we expect to observe a monotonic improvement in visual search performance as we increase the number of combined odd higher harmonics. In contrast, if auditory driven visual search is due to a merely discrete process, then we expect to observe search benefits only in response to the square-wave and do not expect to observe search benefits when we simply add higher odd harmonics to the fundamental sine wave.

**Figure 5:**
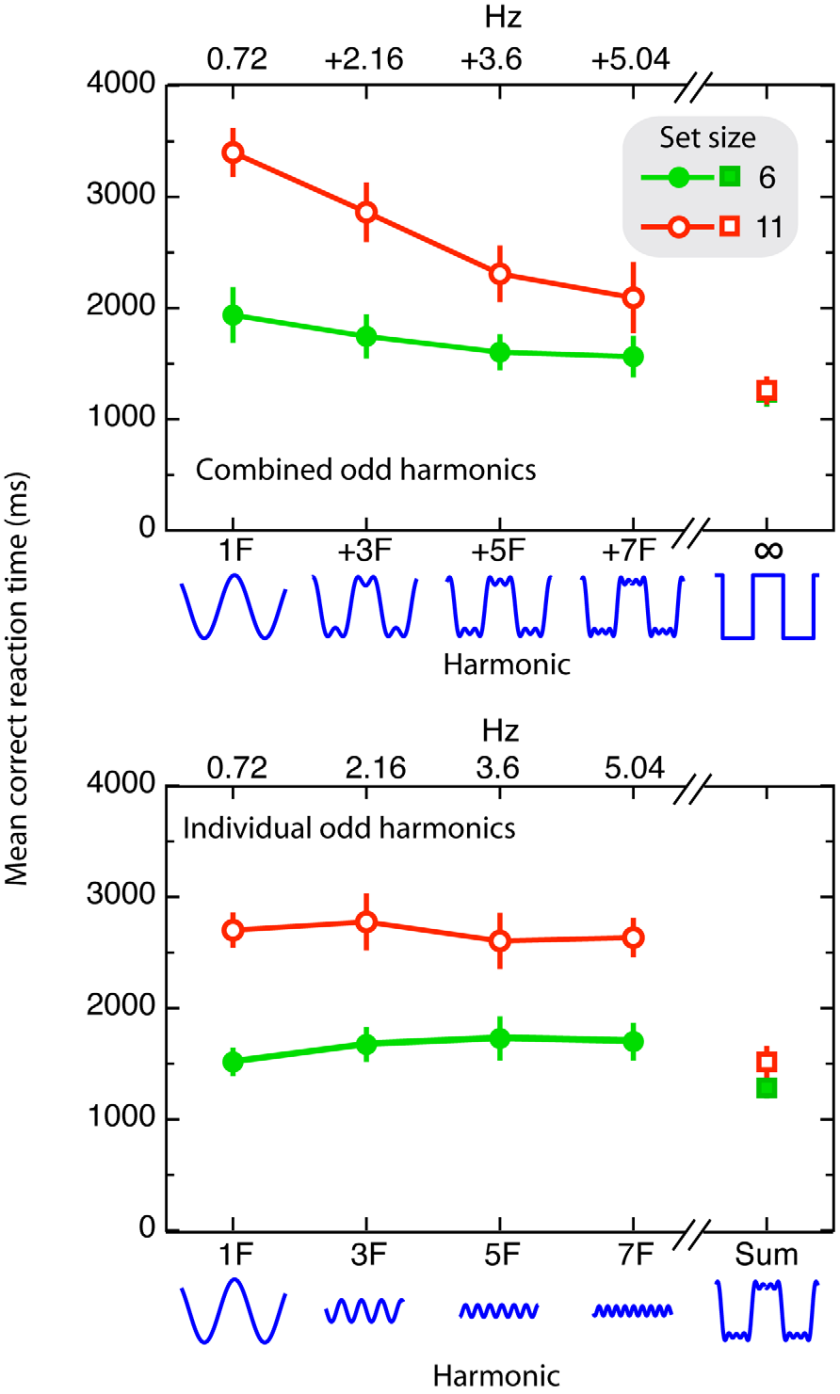
Results of Experiment 3. The upper panel indicates the mean correct reaction time as a function of set size, and stimulus type for each combined odd harmonics. The lower panel indicates the mean correct reaction time as a function of set size, and stimulus type for each individual odd harmonics. The frequency for each individual odd harmonic is plotted on top of the graphs. Overall, adding higher odd harmonics to a fundamental sine-wave increases search efficiency, even though those harmonics alone afford no search benefit.

The results of Experiment 3 are presented in Figure 5. RTs were subjected to an ANOVA with set size (6 vs. 11), and stimulus type [fundamental (F), F+3F, F+3F+5F, F+3F+5F+7F, or square-wave] as within-subject variables. In the control experiment, RTs were subjected to an ANOVA with set size (6 vs. 11), and stimulus type [fundamental (F), 3F, 5F, 7F, or sum of the individuals (F+3F+5F+7F)] as within-subject variables.

Overall mean error rate was 1.3%. The ANOVA revealed a reliable effect of stimulus type, and a significant set size x stimulus type interaction, F(4, 12) = 26.1, p<.001, and F(4, 12) = 20.3, p<.001, respectively. In other words, as is clear from Figure 5, search improved monotonically (in terms of overall RTs and search slopes) as the number of odd harmonics increased (F: 304 ms item^−1^; F+3F: 245 ms item^−1^; F+3F+5F: 168 ms item^−1^; F+3F+5F+7F: 117 ms item^−1^; square-wave: 1 ms item^−1^). However, it is still possible that the observed interaction and main effect of stimulus type were due to the square-wave condition and thus due to a discrete process instead. However, when we excluded the square-wave condition from analyses, we again observed a reliable effect of stimulus type, and a significant set size x stimulus type interaction, F(3, 9) = 36.0, p<.001, and F(3, 9) = 8.0, p<.01, respectively.

In the control experiment (individual harmonics condition), overall mean error rate was 1.2%. The ANOVA revealed a reliable stimulus type effect, F(4, 12) = 16.6, p = .01, as search was significantly faster when the stimulus type contained the sum of the individual harmonics compared to each individual harmonic alone (all *t-values* (3) >4.4, all *p-values*<.02). Moreover, the ANOVA revealed a reliable set size x stimulus type interaction, F(4, 12) = 22.5, p = .001, as the search slope was shallower in the sum of all harmonics condition (49 ms item^−1^) compared to each individual harmonic (F: 252 ms item^−1^; 3F: 231 ms item^−1^; 5F: 190 ms item^−1^; 7F: 199 ms item^−1^). Furthermore, the two-way interaction, and the main effect of stimulus type were not reliable when we excluded the sum of the harmonics condition from the ANOVA, *F*(3, 9) = 5.2, *p* = .09, and *F*<1, *p* = .45, respectively.

The present experiment shows that adding higher odd harmonic frequencies (3F, 5F, and 7F) to a fundamental sine-wave increases search efficiency, although the harmonics alone afford no search benefit at all. The observed search benefits demonstrate that acoustically driven visual search is contingent upon the spectral composition of the audiovisual stimulus, in particular the presence of sharply defined transient changes. There are, however, alternative (and complementary) explanations. For instance, as one adds odd higher harmonics to the fundamental sine wave, the temporal rate of change in stimulus luminance increases monotonically. That is to say, the steepness of the onset and offset increases, becoming more abrupt as the number of harmonics increases. Another possibility is that as one approaches the square-wave (by adding higher harmonics to the sine-wave) the stimulus changes alternate with increasingly static episodes if the target change occurs during a static episode of the distractors search may become more efficient.

### Experiment 4: Distractor dynamics and abrupt transient events

**Figure 6:**
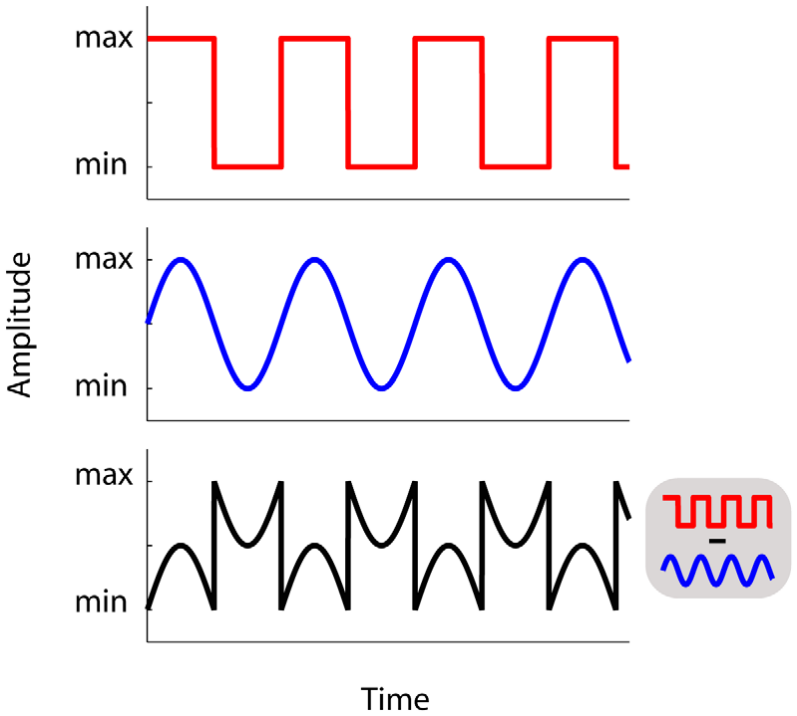
Stimuli used in Experiment 4. The difference-wave (lower panel) reflects the difference between the square- and sine-wave modulations. See Movie S4 for an example difference-wave trial.

To investigate whether the observed search benefits in the previous experiments were due to static distractors in the square-wave condition vs. dynamic distractors in the sine-wave condition we manipulated the distractor dynamics by comparing three different waveforms: sine-wave, square-wave, and difference-wave condition (see Figure 6).

Mathematically, the difference wave (Movie S4; black curve in Figure 6) is the result of a square-wave from which the sine-wave has been subtracted (see grey inset). The difference-wave contains abrupt changes at the same rate as in the square-wave condition, but is otherwise similar in dynamics to the sine-wave condition (since, in effect, it is a series of sinusoidal half-waves separated by abrupt changes). Moreover, the temporal frequency remains unchanged at 0.72 Hz. The experiment was identical to Experiment 2, except that the audiovisual events were always identical. Stimulus type (sine-wave, square-wave vs. difference-wave) was randomly mixed within blocks. Participants received ten experimental blocks of 24 trials, preceded by a single practice block of 24 trials. If search benefits in the square-wave condition are due to the distractors being static at the target change, then we do not expect to observe search benefits in the difference-wave condition. In contrast, if auditory driven search requires steep onset/offset transients, then we expect to observe search benefits in the difference-wave condition as well.

In a control experiment, we investigated whether the steepness, or abruptness, of the audiovisual events is important for auditory driven search. This control experiment was identical to Experiment 2, except that the audiovisual events were always identical. Furthermore, the audiovisual events were synchronized square-wave modulated signals whose onset and offset transitions were manipulated. The onset, and offset steepness was determined by the time to reach the maximum amplitude from the minimum amplitude (following a linear profile), or vice versa. The timing was either 13 ms (i.e. square-wave: an abrupt change from one video frame to the next) or was 27, 53, 80, 106, 160, 266, 399, or 532 ms. Set size was fixed (11 elements). Participants received eight experimental blocks of 36 trials, preceded by a single practice block of 36 trials. If the observed search benefits in the previous experiments are due to the steepness of the audiovisual events, then we expect a monotonic improvement in search performance dependent on the steepness of the onset and offset slope of the audiovisual events.

**Figure 7:**
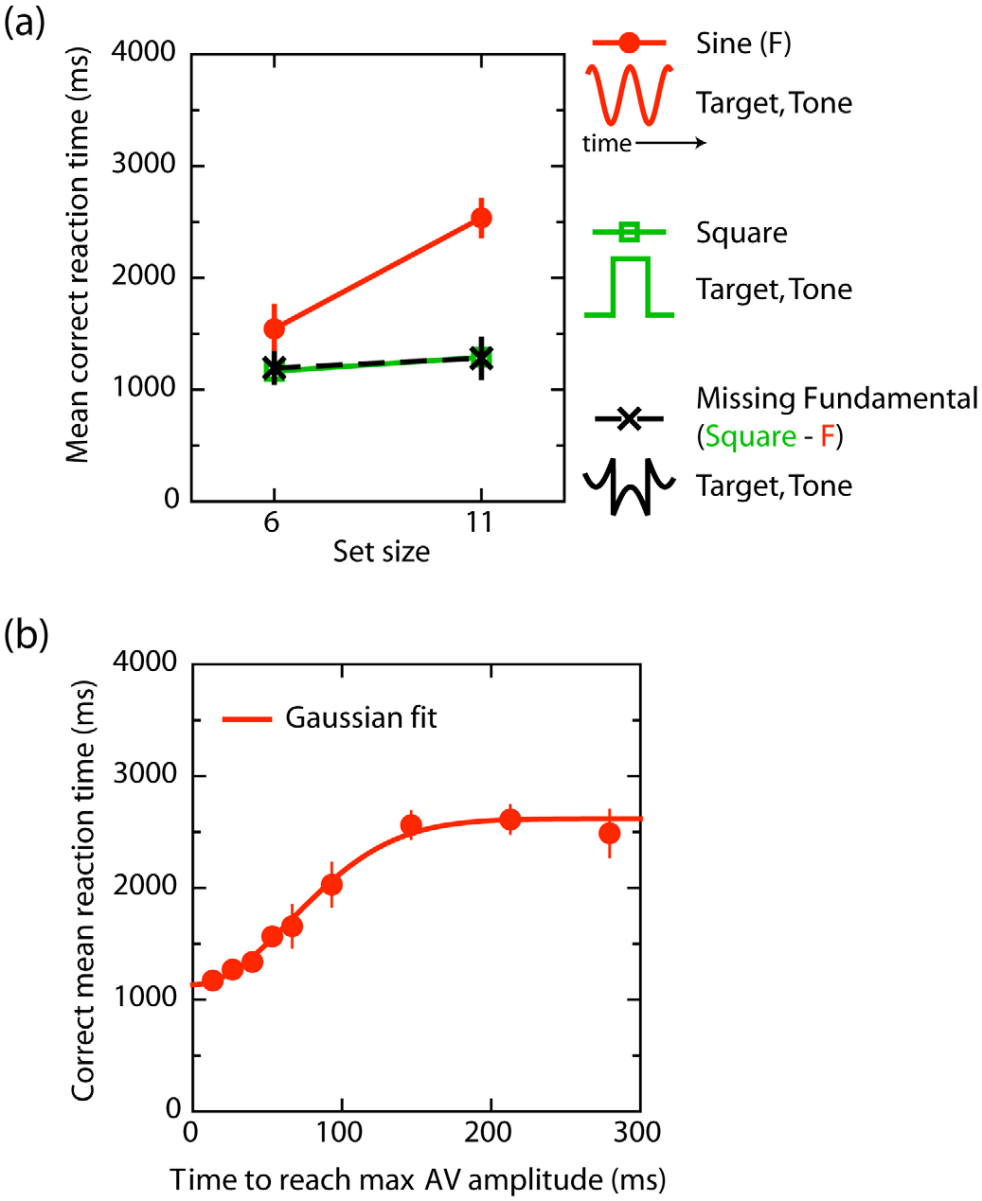
Results of Experiment 4. A) Mean correct reaction time (RT) as a function of set size for each stimulus type. B) Mean correct reaction time (ms) as a function of steepness. The steepness refers to the time to reach the maximum amplitude from its minimum (or vice versa). Note that 13 ms reflects a square-wave modulation. These data show that auditory driven search requires abrupt auditory and visual events.

The results of Experiment 4 are presented in Figure 7. RTs were subjected to an ANOVA with set size (6 vs. 11), and stimulus type (square, sine, or difference-wave) as within-subject variables. In the control experiment, RTs were subjected to an ANOVA with steepness (13, 27, 53, 80, 106, 160, 266, 399, or 532 ms) as within-subject variables.

The overall error rate was 1.2%. The ANOVA revealed a reliable set size x stimulus type interaction, F(2, 6) = 46.4, p<.001. As is clear from Figure 7a, search was much better when the audiovisual events were square-waves (28 ms item^−1^) than when they were sine-waves (199 ms item^−1^), F(1, 3) = 48.4, p<.01. Importantly, however, search was also very good when the audiovisual events were difference-waves (20 ms item^−1^), relative to when the audiovisual events were sine-waves, F(1, 3) = 53.4, p = .005. Indeed, search was equally efficient in the square-wave and difference-wave conditions, in terms of search times, F<1, p>.9, and search slopes, F<1, p>.5. Therefore, even though the difference-wave condition contained similar distractor dynamics as the sine-wave condition while in the square-wave condition the distractors were static during the target color change, search efficiency in these conditions was equivalent.

The results of the control experiment are presented in Figure 7b. RTs were subjected to an ANOVA with steepness (13, 27, 53, 80, 106, 160, 266, 399, or 532 ms) as within-subject variable. The overall error rate was 3.0%. The ANOVA on RTs revealed a reliable effect of steepness, F(8, 24) = 38.1, p<.001, as search monotonically improved as a function of the steepness of intensity change. More specifically, search was optimal when the audiovisual events were square-wave-modulated (1,171 ms; steepness was 13 ms), and search times increased as the steepness reduced. The effect saturated at around 266 ms. The present experiment therefore revealed clear evidence that auditory driven search requires abrupt auditory and visual events.

## Discussion

To recap, the aim of this study was to examine what information is critical for a non-spatial acoustic signal to drive efficient visual search. We observed that audiovisual square-wave modulations that were synchronised in time facilitate visual search (Experiments 1–4). In contrast, visual search is not facilitated when audiovisual signals with the same frequency and temporal phase modulate sinusoidally (Experiments 1–4). Experiment 2 showed that this acoustically driven visual search efficiency requires square-wave luminance modulations in vision, although auditory events can be sinusoidal. We also show that visual search efficiency improves monotonically with the number of odd harmonics added to the fundamental sine-wave (progressively approximating a square-wave), although the harmonics presented alone afford no search benefit (Experiment 3). Furthermore, we show that these search benefits are not due to the dynamic properties of the surrounding distractors, but due to an increase in audiovisual onset and/or offset slope (Experiment 4).

Whereas square-wave audiovisual modulations improved the efficiency of spatial visual search, audiovisual sine-wave modulations did not, even though the fundamental temporal frequency (0.72 Hz) was identical in both situations, and sufficiently low (< 4Hz) for audiovisual interaction to occur [23], [24]. The present study appear at odds with a study by Fujisaki et al. [15], who also looked at the influence of auditory signals on visual search. In their study, participants were asked to detect a flashing or rotating visual target amongst a number of flashing or rotating distractors. The target dynamics were either accompanied by a brief “pip” or by frequency modulated sweeps. The presence of these sounds did not result in search benefits whatsoever. In Experiment 4, we showed that auditory driven visual search requires steep, abrupt audiovisual events. This explains why Fujisaki et al. were not able to find search benefits when they used a continuous changing rotating visual target in combination with a synchronized frequency modulated auditory signal (i.e., a fact that we confirmed in several sine-wave conditions; Experiments 1–4). But why were Fujisaki et al. not able to find search benefits when they used abrupt audiovisual events?

An important question is whether the rate of abrupt audiovisual events would be an important factor for auditory driven visual search. For instance, if one increases the rate at which abrupt audiovisual events occur, this will result in a decreasing temporal separation between the target and distractor annuli. This might be harmful for auditory driven visual search because it is known that audiovisual synchrony has a very wide temporal window of integration (see Figure S1, as well as several other studies[16], [25]) and therefore an increased probability of integrating distractors and target at higher temporal rates. As a consequence, distractor events might affect optimal audiovisual performance [26], and could even end up capturing attention [16], [27]. In Experiment S2, we manipulated the minimal temporal separation between the target and distractor annuli, while the auditory signal was always synchronized with the target annulus. The data (see Figure S2) confirmed that when the temporal separation between the target and distractor annuli was greater than 120 ms, auditory driven search was highly efficient. In contrast, search benefits were no longer efficient when the minimal temporal separation between the target and distractor annuli was smaller than 120 ms, even though the auditory signal was always synchronized with the visual target annulus. This finding is consistent with previous studies reporting audiovisual benefits when the temporal separation between the target and distractor events is greater than 120 ms [7], [10], [16], [18], and no effect of multisensory interaction in the case of possible temporal overlap [15]. The minimal temporal separation of 120 ms indicates that a distractor change should not occur within a temporal window of 240 ms (120 ms prior to the target event, and 120 ms after the target event), a temporal period corresponding to a frequency of 4.1 Hz. It is important to note that because auditory-driven visual search requires abrupt audiovisual changes, this frequency does *not* indicate the maximum frequency of modulation but the maximum number of abrupt events per second (irrespective of whether they are onsets or offsets). This also explains why Fujisaki et al. were not able to find search benefits when they used abrupt audiovisual events because the temporal frequency was too high (up to 40 Hz), and they did not control for temporal overlap.

An intriguing question is where and when in the brain this transient audiovisual synchrony process occurs. Although there are numerous areas in the brain containing multisensory neurons ([see e.g. 28], [29], for reviews), they are particularly abundant in the superior colliculus (SC)[4], [30]. Consistent with the present study (see e.g. Figure S1 online), this midbrain structure is responsive to the synchrony of multisensory events, and especially so when an auditory signal follows a visual signal [31]. However, strong activation in SC is only observed when the signals from the different senses arrive from overlapping spatial locations. If the two signals are not spatially collocated activation in SC is greatly reduced or even inhibited[30]. Therefore, SC is unlikely to be the underlying mechanism of the present findings because our temporally aligned multisensory events never shared the same spatial location (see also [16], [18], [27]).

A more likely substrate for the findings we observe would be an early activation in the superior temporal sulcus (STS). There are several good reasons for this. First, the STS has been shown to be sensitive to the synchronization of audiovisual events [32], [33], [34]. Second, whereas these studies reported synchrony effects in the STS when using semantic information, other studies found similar effects when using synchronized non-semantic flashes in combination with simple beeps [35], [36]. Third, in contrast to the SC, these multisensory interactions were observed even though the auditory and visual stimuli were never spatially aligned [32], [36]. Finally, it is known that the STS forms part of the visual cortex [37], and therefore an auditory signal could, through cortical feedback, affect perceptual processing of a synchronized visual event at an early level of processing [38]. As a result, the synchronized visual event is perceived as being brighter [39] and therefore pops-out from its environment.

The present study highlights the importance of transients in audiovisual interaction. There is great potential for false matches in either spatial and/or temporal audiovisual integration. Spatially, events must come from approximately the same location to be integrated. However, as is observed in the phenomenon known as ventriloquism, false matches may occur when the locations of multisensory events are uncertain [6], [11]. Similarly in the time domain, to avoid false matches, events must be synchronous. However, we have shown that temporal alignment alone is not sufficient for target-specific audiovisual binding to occur. If temporal alignment was all that mattered, the synchronized sine-wave modulations would have produced efficient visual search, yet this was not observed. Rather, audiovisual interaction required temporal alignment of *transient* audiovisual events. Why transient? Transient events are, by definition, brief. By integrating only transient events defined very narrowly in time, the possibility of spuriously interacting unrelated auditory and visual events within the broad temporal window of multisensory interaction is greatly reduced (see e.g. Figure S1 as well as [16], [25]). In line with this reasoning, transient visual information has been found to be critical for perceptually differentiating events across time [40]. The present results emphasize the importance of abrupt events in interacting multimodal temporal information in natural scenes.

## Materials and Methods

### Ethics Statement

Written consent was obtained from each participant prior to the experiments. The experiments were approved by the local ethics committee of the University of Sydney.

### Participants

Four participants (two naïve) took part in all experiments (2 female, mean age 30.2 years; range 24–37).

### Stimuli and apparatus

Experiments were run in a dimly lit, air-conditioned room. Participants were seated at approximately 80 cm from the monitor (75 Hz refresh rate). The visual search displays consisted of six or eleven white line segments (116.5 cd m^−2^, length 0.7° visual angle) on a black (<0.5 cd m^−2^) background. All lines were equally spaced and randomly placed on an imaginary circle (4.9° radius) centered on a white fixation dot (116.5 cd m^−2^). The orientation of each line deviated randomly by either plus or minus 3° from horizontal or vertical, except for the target which was horizontal or vertical. Each line segment was surrounded by a grey annulus (1.1° radius, 0.4° width), whose luminance varied across frames (13.3 ms per frame). The luminance range for each annulus varied between 8.9 and 68.8 cd m^−2^. On half of the trials the luminance of the visual annuli followed a sine-wave with a temporal frequency of 0.72 Hz, and on the remaining trials the luminance of the visual annuli followed a square-wave modulation profile (0.72 Hz). The phase between the target annulus (i.e. the one containing the horizontal or vertical line segment) and the remaining distractor annuli was controlled to avoid overlapping temporal synchrony (±50°, 70°, 90°, 110°or 310° out of phase). In this way, the phase differences resulted in a minimal temporal separation of 200 ms (i.e. 80 ms greater than the critical temporal separation of 120 ms between the onset/offset of target and distractor modulation; see Experiment S2 online).

The auditory stimulus was a 500 Hz sine-wave (44.1 kHz sample rate; 16 bit; mono) and presented via a loudspeaker, which was placed above the monitor. This auditory signal was modulated in the amplitude domain and varied in level between 42 and 76 dB. On half of the trials, the amplitude followed a square-wave pattern, and on the remaining trials it followed a sine-wave pattern. The fundamental frequency of the sine- and square-wave modulators was o.72 Hz. If present, the auditory signal's modulation always matched the target annulus (i.e. the audiovisual events were both sine-waves, or both square-waves). Moreover, on half of these trials, the auditory signal was completely synchronized with the target annulus (i.e. 0° phase shift between the auditory and visual signal), and on the other half the auditory signal was out of phase (i.e. 180° phase shift between the auditory and visual signal). Participants were aware that the auditory signal, if present, was always synchronized with the target annulus.

### Design and procedure

On any given trial the total number of target plus distractor items presented (set size) was 6 or 11. The audiovisual stimulus type was either sine-wave or square-wave modulated. The other manipulation involved the presentation of a tone coinciding with the target [tone present (in phase), tone present (180° out of phase), and tone absent]. Dependent variables were the reaction time (RT) and accuracy to detect the target. Note that the RT reflects the time between the search display onset and the response to the target, because the target was present when the search display appeared. Each trial began with a fixation dot presented for 1,000 ms at the center of the screen. The search display was presented until participants responded. Participants were asked to remain fixated on the fixation dot, and instructed to press the z- or m-key on the standard keyboard as fast and accurately as possible when the target orientation was horizontal or vertical, respectively. Target orientation was balanced and randomly mixed within blocks of 32 trials each. Tone presence and stimulus type were randomly mixed within blocks. Participants received ten experimental blocks, preceded by one practice block. Participants received feedback about their overall mean accuracy and overall mean RT after each block.

## Supporting Information

Experiment S1 Details Experiment S1.
(0.04 MB DOC)

Experiment S2 Details Experiment S2.
(0.03 MB DOC)

Figure S1 Results of Experiment S1. Correct mean reaction time as a function of stimulus type and tone target interval (TTI). Note that negative TTI's indicate that the tone preceded the visual event and that positive TTI's indicate that the tone followed the visual target event. The set size was always fixed (11 items).
(3.55 MB TIF)

Figure S2 Results of Experiment S2. Correct mean reaction time as a function of set size, and temporal separation.
(3.54 MB TIF)

Movie S1 Example trial in Experiment 1. In this example, the audiovisual events were both sinusoidal modulated with a frequency of 0.72 Hz. The tone is synchronized (0°out of phase) with the target annulus (i.e. the one containing the horizontal or vertical line segment). Note that the frequency of the modulation is dependent on the refresh-rate of your computer.
(1.85 MB MOV)

Movie S2 Example trial in Experiment 1. In this example, the audiovisual events were both square-wave modulated with a frequency of 0.72 Hz. The tone is synchronized (0°out of phase) with the target annulus.
(1.82 MB MOV)

Movie S3 Example trial in Experiment 3. In this example, the audiovisual events were the summation of the fundamental frequency (F; i.e. the sine-wave condition in Mov. S1), the third higher harmonic (3F) and the fifth higher harmonic (5F).
(2.21 MB MOV)

Movie S4 Example trial in Experiment 4. In this example, the audiovisual events represent difference-wave condition, with a frequency of 0.72 Hz.
(1.79 MB MOV)
