# Supplementary material for: Efficient Visual Search from Synchronized Auditory Signals Requires Transient Audiovisual Events
Source: PLoS One. 2010 May 14;5(5):e10664. doi: 10.1371/journal.pone.0010664 (PMC2871056; doi:10.1371/journal.pone.0010664)
Supplement: Experiment S1 — Details Experiment S1. (0.04 MB DOC) [file pone.0010664.s001.doc]

**Experiment S1**

**Methods**

The experiment was identical to Experiment 1, except that the tone was always present and synchronized with the target annulus (i.e. 0˚ out of phase), but not necessarily synchronized with the visual target annulus. The tone target interval (TTI; see also Van der Burg et al., 2008) varied between -160, -80, -40, 0, 40, 80, 160 ms. Furthermore, set size was fixed at 11. Stimulus type (sine- vs. square-wave) and TTI (-160, -80, -40, 0, 40, 80, or 160 ms) were randomly mixed within blocks. Participants participated in two sessions. One session contained one practice block and ten experimental blocks of 56 trials each.

**Results**

The results are presented in **Fig. S1**. RTs were subjected to an ANOVA with stimulus type (sine- vs. square-wave), and TTI (-160, -80, -40, 0, 40, 80, or 160 ms) as within-subject variables.

Overall mean error rate was 2.1%. Consistent with Experiment 1, responses were overall significantly faster when the audiovisual events were square-waves (1,470 ms) than when the audiovisual events were sine-waves (2,742 ms), *F*(1, 3) = 14.8, *p* < .05. Furthermore, the ANOVA on RTs revealed a reliable stimulus type x TTI interaction, *F*(6, 18) = 3.5, *p* = .018. This interaction was further examined by separate ANOVAs for each stimulus type. The TTI manipulation had an enormous effect on search performance when the audiovisual events were square-wave modulated, F(6, 18) = 16.7, p < .001, and search performance was optimal when the tone followed the visual target annuli by 40 ms (Lewald et al., 2003; Senkowski et al., 2007; Jaskowski et al., 1990; Wallace et al., 1996). In contrast, the TTI manipulation had no effect on search performance when the audiovisual events were sine-wave modulated, *F*(6, 18) = 2.0, *p* = .128, and search performance was optimal when the visual target annulus followed the tone by 80 ms.
