# Supplementary material for: Efficient Visual Search from Synchronized Auditory Signals Requires Transient Audiovisual Events
Source: PLoS One. 2010 May 14;5(5):e10664. doi: 10.1371/journal.pone.0010664 (PMC2871056; doi:10.1371/journal.pone.0010664)
Supplement: Experiment S2 — Details Experiment S2. (0.03 MB DOC) [file pone.0010664.s002.doc]

**Experiment S2**

**Methods**

The experiment was identical to Experiment 1, except that the tone was always present and synchronized with the target annulus (i.e. 0˚ out of phase), but the minimal temporal separation between the target and distractor annuli was not necessarily 200 ms (± 50˚ out of phase). In the present experiment we manipulated the minimal phase between the target annulus and thus the minimal temporal separation between the target and distractor annuli (13, 40, 67, 93, 120, 147, 173, or 200 ms). Furthermore, the audiovisual events were always square-wave modulated (0.72 Hz). Set size (6 vs. 11) and temporal separation 13, 40, 67, 93, 120, 147, 173, or 200 ms) were randomly mixed within blocks of 32 trials each. Participants participated in two sessions. One session contained one practice block and ten experimental blocks.

**Results**

The results are presented in **Fig. S2**. RTs were subjected to an ANOVA with set size (6 vs. 11), and temporal separation 13, 40, 67, 93, 120, 147, 173, or 200 ms) as within-subject variables.

Overall mean error rate was 2.5%. There was a reliable temporal separation effect, *F*(7, 21) = 55.8, *p* < .001, as the overall search times decreased as the temporal separation between the target and distractor annuli increased. Furthermore, the temporal separation x set size interaction was reliable, *F*(7, 21) = 35.9, *p* < .001, as the search slope decreased as the temporal separation increased. The search slope effects were analyzed by separate two-tailed t-test for each temporal separation compared to a temporal separation of 200 ms. When the temporal separation was < 120 ms, the analyses yielded a reliable difference compared to the 200 ms condition, *ts* > 8.5, *ps* < .005. In contrast, no such a difference was reliable when the temporal separation was ≥ 120 ms compared to the 200 ms condition, all *ts* < 2.9, *p* > .05.
