# Supplementary figures and images for: Efficient Visual Search from Synchronized Auditory Signals Requires Transient Audiovisual Events
Source: PLoS One. 2010 May 14;5(5):e10664. doi: 10.1371/journal.pone.0010664 (PMC2871056; doi:10.1371/journal.pone.0010664)

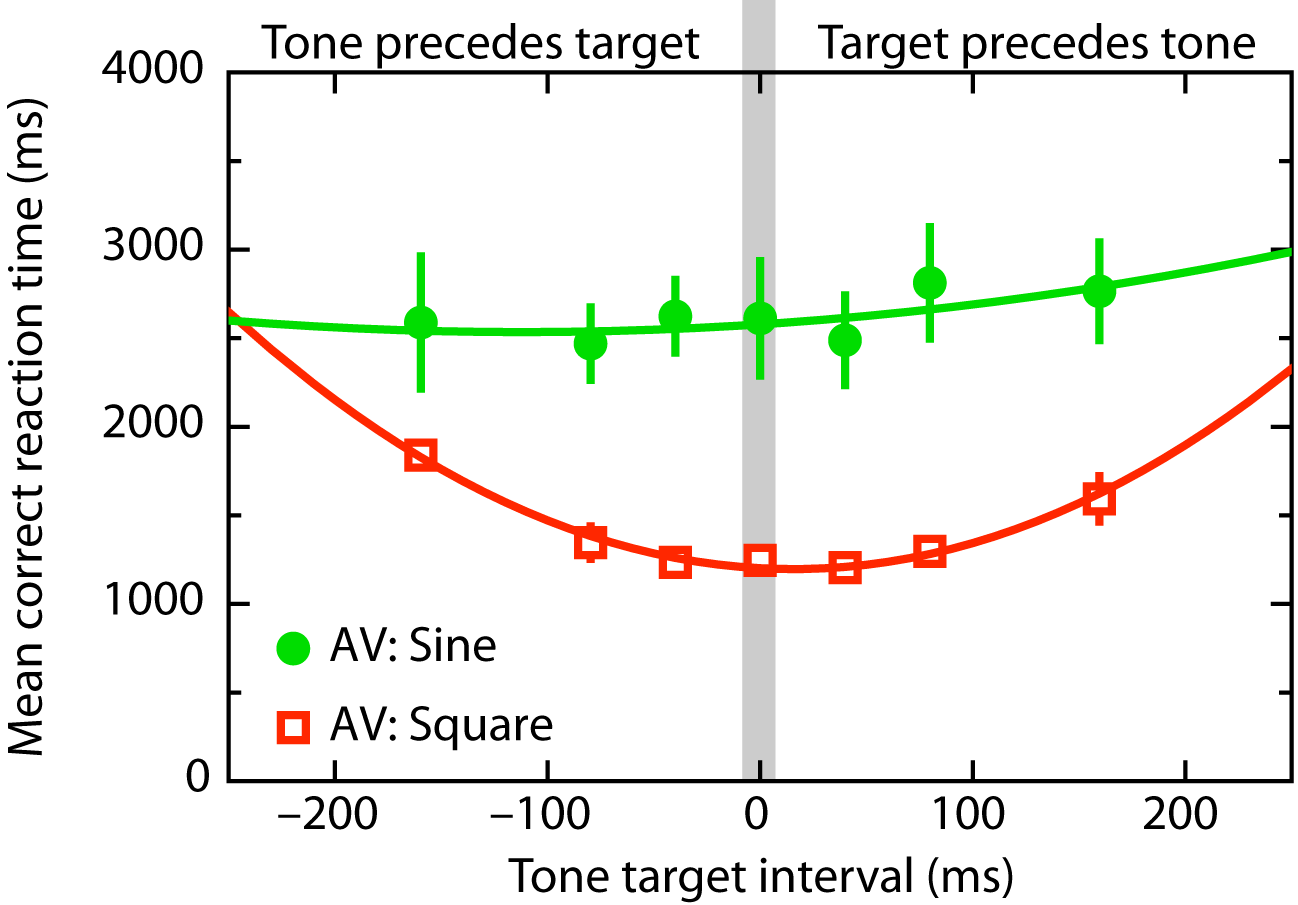

Supplement: Figure S1 — Results of Experiment S1. Correct mean reaction time as a function of stimulus type and tone target interval (TTI). Note that negative TTI's indicate that the tone preceded the visual event and that positive TTI's indicate that the tone followed the visual target event. The set size was always fixed (11 items). (3.55 MB TIF) [file pone.0010664.s003.tif]

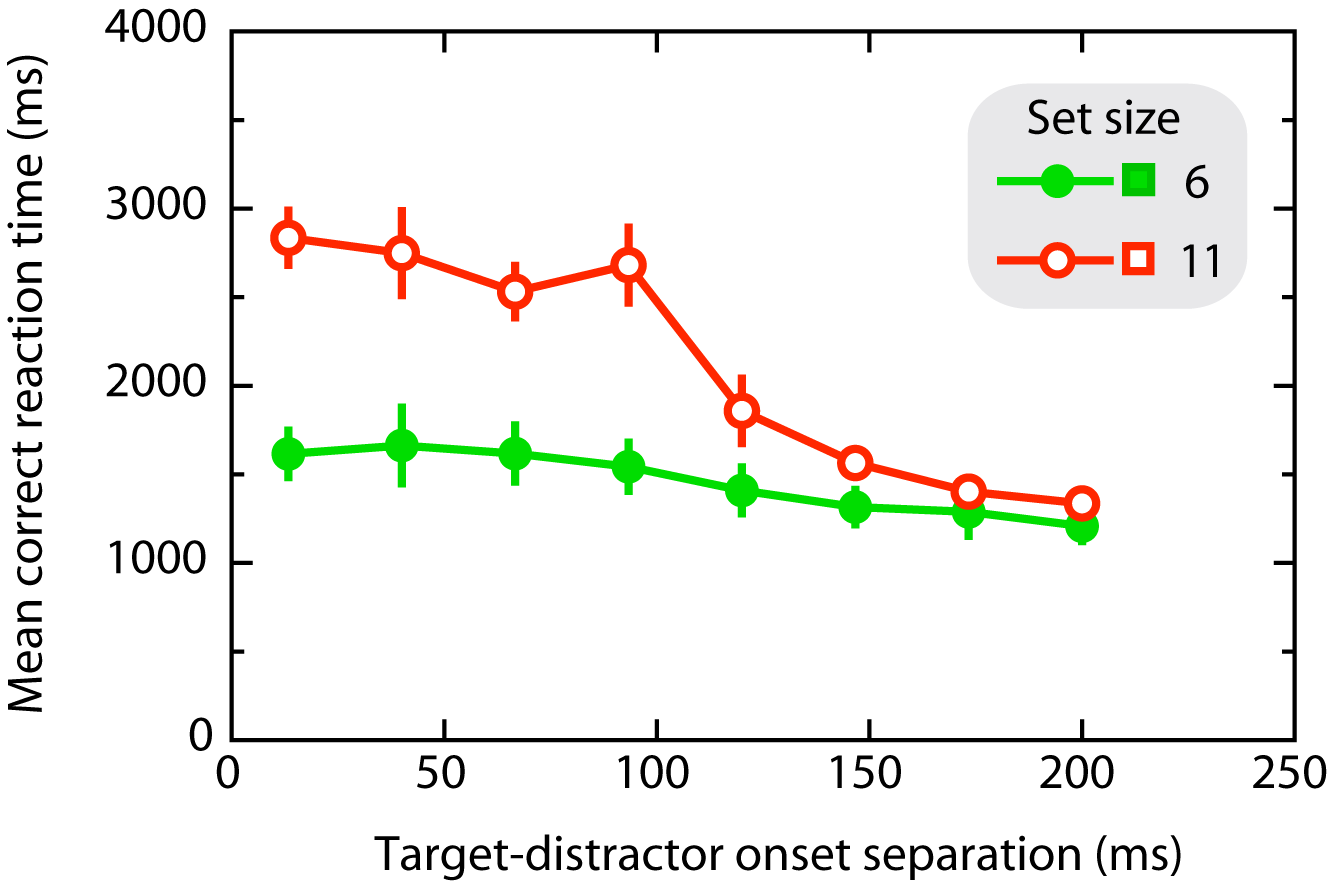

Supplement: Figure S2 — Results of Experiment S2. Correct mean reaction time as a function of set size, and temporal separation. (3.54 MB TIF) [file pone.0010664.s004.tif]
